# Supplementary material for: Splice-Junction-Based Mapping of Alternative Isoforms in the Human Proteome
Source: Cell Rep. Author manuscript; Available in PMC 2020 Jan 15. (PMC6961840; doi:10.1016/j.celrep.2019.11.026)
Supplement: 2 [file NIHMS1546469-supplement-2.zip › DF1/PXD010154/Prostate/Prostate_3_CLTCL1_LQEHFQLQNLGINPANIGFSTLTMESDK.pdf]

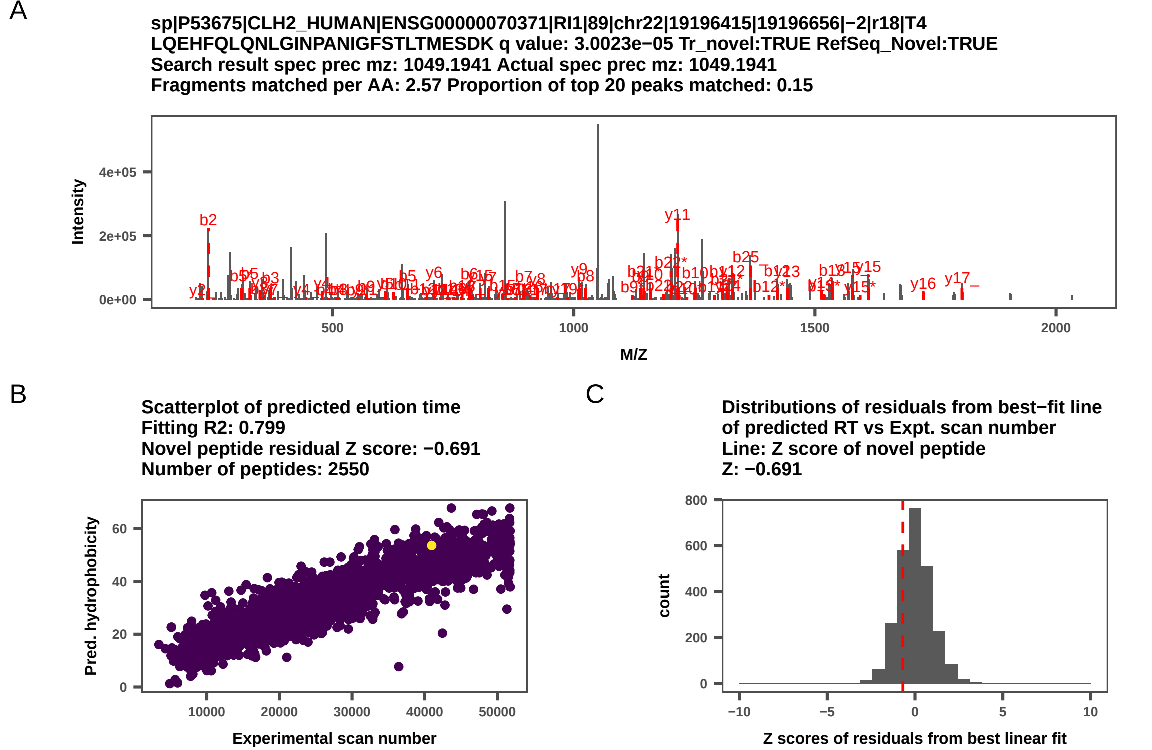
A
sp|P53675|CLH2_HUMAN|ENSG00000070371|RI1|89|chr22|19196415|19196656|−2|r18|T4
LQEHFQLQNLGINPANIGFSTLTMESDK q value: 3.0023e−05 Tr_novel:TRUE RefSeq_Novel:TRUE
Search result spec prec mz: 1049.1941 Actual spec prec mz: 1049.1941
Fragments matched per AA: 2.57 Proportion of top 20 peaks matched: 0.15
Intensity
4e+05
2e+05
0e+00
M/Z
500
1000
1500
2000
b2
y11
b25
y16
y17
B
Scatterplot of predicted elution time
Fitting R2: 0.799
Novel peptide residual Z score: −0.691
Number of peptides: 2550
Pred. hydrophobicity
60
40
20
0
10000
20000
30000
40000
50000
Experimental scan number
C
Distributions of residuals from best−fit line
of predicted RT vs Expt. scan number
Line: Z score of novel peptide
Z: −0.691
count
800
600
400
200
0
−10
−5
0
5
10
Z scores of residuals from best linear fit
